# Obesity: The Fat Tissue Disease Version of Cancer

**DOI:** 10.3390/cells11121872

**Published:** 2022-06-09

**Authors:** Besma Boubertakh, Cristoforo Silvestri, Vincenzo Di Marzo

**Affiliations:** 1 Centre de Recherche de l’Institut Universitaire de Cardiologie et de Pneumologie de Québec, Département of Médecine, Faculté de Médecine, Université Laval, Québec City, QC G1V 0A6, Canada; cristoforo.silvestri@criucpq.ulaval.ca; 2 Canada Excellence Research Chair on the Microbiome-Endocannabinoidome Axis in Metabolic Health (CERC-MEND), Université Laval, Québec City, QC G1V 0A6, Canada; 3 Endocannabinoid Research Group, Institute of Biomolecular Chemistry, Consiglio Nazionale Delle Ricerche (CNR), 80078 Pozzuoli, Italy; 4 Institut sur la Nutrition et les Aliments Fonctionnels, Centre NUTRISS, École de Nutrition, Faculté des Sciences de L’agriculture et de L’alimentation, Université Laval, Québec City, QC G1V 0A6, Canada; 5 Joint International Unit between the Consiglio Nazionale Delle Ricerche (Italy) and Université Laval (Canada) on Chemical and Biomolecular Research on the Microbiome and Its Impact on Metabolic Health and Nutrition (UMI-MicroMeNu), 80078 Pozzuoli, Italy and Québec City, QC G1V 0A6, Canada

**Keywords:** obesity, disease, cancer, white adipose tissue, cell growth, cell proliferation, metastasis, angiogenesis, recurrence, gut microbiome

## Abstract

Obesity is a disease with high potential for fatality. It perfectly fits the disease definition, as cancer does. This is because it damages body structure and functions, both mechanically and biologically, and alters physical, mental, and social health. In addition, it shares many common morbid characteristics with the most feared disease, cancer. For example, it is influenced by a sophisticated interaction between a person’s genetics, the environment, and an increasing number of other backgrounds. Furthermore, it displays abnormal cell growth and proliferation events, only limited to white fat, resulting in adipose tissue taking up an increasing amount of space within the body. This occurs through fat “metastases” and via altered signaling that further aggravates the pathology of obesity by inducing ubiquitous dishomeostasis. These metastases can be made graver by angiogenesis, which might boost diseased tissue growth. More common features with cancer include its progressive escalation through different levels of severity and its possibility of re-onset after recovery. Despite all these similarities with cancer, obesity is substantially less agitating for most people. Thus, the ideas proposed herein could have utility to sensitize the public opinion about the hard reality of obesity. This is increasingly needed, as the obesity pandemic has waged a fierce war against our bodies and society in general, while there is still doubt about whether it is a real disease or not. Hence, raising public consciousness to properly face health issues is crucial to improving our health instead of gaining weight unhealthily. It is obviously illogical to fight cancer extremely seriously on the one hand and to consider dying with obesity as self-inflicted on the other. In fact, obesity merits a top position among the most lethal diseases besides cancer.

## 1. Introduction

In our initial steps of perceiving life, we assimilate the names and categories of our surroundings, and we address them accordingly. Clearly, if we consider an apple to be a book, it will decompose after a few days on the bookshelf. Likewise, if we see a disease as a simple discomforting “dis-ease”, we might accelerate the extinction of a healthy humankind, especially if we do not identify a hazardous disease, such as obesity. Unfortunately, although obesity has been recognized as a disease by the World Health Organization (WHO) since 1948, followed by an increasing number of organizations worldwide, “globesity” has been undervalued, as Carol Condon emphasizes in the review paper rightly entitled “The fat bomb exploded but no one heard the bang” [1].

Erroneously, fatness is still considered a sign of wealth or beauty in some low-income countries, such as in some regions of the continent of one of us authors, Africa. Conversely, while being wealthier and having more access to health education, the populations of more industrialized countries still mostly show ambiguity regarding the idea that corpulence is a disease or a risk factor in many diseases. Globally, health insurance companies could have enhanced such unawareness through the refusal to recognize obesity as a disease. Moreover, the marketing strategies of potentially obesogenic food and beverage industries that neglect sensitization about the implications in obesity of their products could have magnified this unconsciousness [2]. Indeed, we encounter such products with attractive shapes, colors, and flavors everywhere, but they are never labeled with photos of things such as steatotic liver or atherosclerotic plaque, for example. In contrast, we commonly see photos of throat or lung cancer, or other smoking-related types of cancer, on cigarette packets, which have been shown to effectively alter public opinions about smoking [3,4]. In addition to all this, social media usually wrongly presents obesity as a mere issue of appearance, thus further boosting the general under-appreciation of this very serious clinical problem [5].

Obesity is a deadly global pandemic, as about 3.4 million obese patients and overweight individuals die yearly [6,7]. Additionally, it contributes to the onset, pervasiveness, severity, and fatality of other illnesses, such as osteoarthritis [8], non-alcoholic fatty liver disease [9], and obstructive sleep apnea [10]; it is also associated with the three leading causes of death, namely, heart disease [11], cancer [12] and—more recently—coronavirus disease [13], by affecting metabolism and different body systems, such as the immune system, and engendering a chronic low-grade inflammation that accelerates several other disorders [14,15]. Nonetheless, reluctance still exists regarding its labeling as a disease, with assertions that such recognition might render obese patients unwilling to make efforts to ameliorate their health [16]. However, such assumptions might be rather subjective, because they are not based on the inherent characteristics of what a disease is. Generally, many people are misled by believing that obesity solely adds extra X letters to the person’s clothing size, which could be exactly the same size as a robust person. Such a shallow view might blur the dangerous X letter that could be developing underneath, through the “metabolic syndrome X” [17], which could be gradually displacing tick marks from physiological towards pathological patterns, such as insulin resistance, dyslipidemia and hypertension [18]. On the other hand, this view also neglects the increasing realization that some types of moderate obesity (with a body mass index (BMI) around 30–32 kg/m^2^)—while being visually unpleasant for some—can be still devoid of health complications, whereas others—such as abdominal obesity (a condition that may also appear in individuals with 25 kg/m^2^ <BMI <30 kg/m^2^), which is still socially acceptable—can be accompanied by cardiometabolic risk as much as morbid obesity [19,20].

Being a contributor to multiple other sicknesses, including stroke, myocardial infarction, and cancer, could have led to the fallacious consideration of obesity as a mere risk factor, while it is one of the most dangerous illnesses. By contrast, there is a worldwide consensus that cancer is the most frightening of sicknesses [21]. Since obesity is a disease and a common cause of many other fatal illnesses, such misconception should be addressed urgently. Indeed, this is the objective of this piece of writing, which aims at depicting obesity morbidity and the numerous pathological attributes that it has in common with the worldwide number-one feared disease, cancer.

## 2. Obesity Is a Disease

It is widely agreed upon that cancer is a disease, and there is no rational reason that this does not apply to every other sickness. Obviously, we should not disregard obesity, which is itself a disease and contributes to the onset and progression of cancer and other lethal morbi [12]. Even though the definitions of disease and obesity and whether obesity is a morbus, are continually disputed [22,23], an international consensus based on mostly agreed-upon definitions should be established to circumvent such ambiguities. Certain claims support identifying obesity as a disease, as this might help destigmatize obese patients [23]. Although such stigmatization should undoubtedly be avoided, this should be achieved through proper education about the importance of respecting everyone, regardless of their status. Indeed, regardless of the positive outcomes that might result from labeling obesity as a disease, obesity’s morbus identity possesses objective merit based on solidly established definitions [24,25,26,27,28].

The Stedman’s Medical Dictionary introduces “disease” as a synonym for “illness”, “morbus”, and “sickness”, and as “1. An interruption, cessation, or disorder of a body, system, or organ structure or function. 2. A morbid entity ordinarily characterized by two or more of the following criteria: recognized etiologic agent(s), identifiable group of signs and symptoms, or consistent anatomic alterations”. Similarly, the Oxford Concise Medical Dictionary defines it as “a disorder with a specific cause (which may or may not be known) and recognizable signs and symptoms; any bodily abnormality or failure to function properly, except that resulting directly from physical injury (the latter, however, may open the way for disease)”. The WHO, on the other pan of the scales, presents obesity as an anomalous or excessive stockpile of body fat that poses a risk to the health [29], which is determined by a BMI that is greater than or equal to 30 kg/m^2^, a diagnosis criterion cutoff that could vary according to ethnic groups and other physiological statuses. For example, the BMI value can be healthily higher in the case of a highly muscular or a pregnant body.

By comparing the two concepts, obesity fits perfectly within the definition of “disease”. Indeed, patients with obesity witness a plethora of health disorders and present recognizable pathological manifestations. Mechanically, obesity imposes an excessive pressure on the bones and the joints besides potentially causing airway obstruction that can lead to obstructive sleep apnea [10]. Biologically, it can lead to osteoarthritis [30], and it presents with inflammatory and dysregulated adipokine levels secreted by dysfunctional adipocytes [31,32] in addition to the ectopic abnormal deposition of white adipose tissue (WAT) on vital organs, which seriously alters whole-organism homeostasis [33].

Although relying on the BMI criterion to diagnose obesity is usually controversial [34], as certain individuals can be metabolically healthy at a BMI > 30 kg/m^2^, such individuals still suffer from the symptoms of mechanical origin, with a few exceptions, such as individuals who present a high amount of muscle that supports the knees [35]. For example, sumo wrestlers are mostly metabolically healthy despite their extremely high BMI because of their intensive physical exercise that keeps them fit and fat, yet they suffer from obesity-induced mechanic disorders [36]. However, BMI through the sole measuring of the body’s height and weight cannot properly reflect the metabolic dysregulation and should be supported by waist girth, blood triglyceride levels, other measurements that can reflect abdominal (visceral) obesity, which is highly morbid [37].

In fact, patients with obesity may find sundry difficulties in accomplishing simple daily life activities, such as tying their shoelaces, which can affect their psychological health [38]. This is further worsened by social media’s deep influence through promoting an “ideal” unrealistic body shape image, which stigmatizes patients with obesity and erroneously makes them feel like incompetent humans [5] in addition to presenting deceptive advice about losing weight strategies [39]. Overall, obesity presents deteriorations in all levels of health, which is defined by the WHO as “a state of complete physical, mental and social well-being and not merely the absence of disease and infirmity” [40]. We can envision this through the difficulty we experience during snowy days when we find it difficult to put on our shoes after wearing a heavy winter jacket, not to mention the suffering of obesity patients. Considering the definitions of obesity, disease, and health, obesity is indisputably a disease.

## 3. The Obesity–Cancer Connection: Exemplification of Potential Disease Amplification

Obesity’s severity is further aggravated by its capacity to trigger, advance, or favor poor prognosis of, other illnesses through what we might call “obesity indirect downstream metastases”. We are suggesting this expression in reference to the ability of affected WAT alterations in obesity to “metastasize” by inducing or worsening health status with respect to other morbi in other organs. This can be potentially life-threatening, particularly in the case of cancer, which is per se metastatic.

The obesity–cancer causal connection has been extensively hypothesized, investigated, evidenced, and reviewed [41,42,43,44,45,46]. This has built a growing body of consensus about the association of these two pathologies, besides their broadly proven correlation, given the numerous epidemiological and clinical studies pointing out the significantly higher relative risk to develop cancer in obese versus non-obese populations [47,48]. Explorations about these connecting bridges have yielded promising answers, such as the recognition of fat mass and obesity-associated genes (e.g., *FTO*) as a common mechanistic basis for both cancer and obesity and the finding that obesity-associated dysmetabolism causes genotoxic stress in favor of cancer comorbidity [49,50,51]. This evidence adds to the results from many other in vitro [51,52,53], ex vivo [54,55], and in vivo studies [56,57,58,59] and has been reviewed and acknowledged by the International Agency for Research on Cancer, who announced that there is enough confident and unbiased evidence about the association of excess body weight with a reinforced cancer predisposition—in particular with regard to more than twelve types of cancer in various tissues/organ systems, such as blood, central nervous system, endometrium, esophagus, kidney, pancreas, liver, colon, postmenopausal breast, ovary, gallbladder, and thyroid gland—in agreement with the World Cancer Research Fund/American Institute for Cancer Research [58,60].

Importantly, not only are most WAT cell types potential tumor triggers and promoters, but mature adipocytes might further undergo dedifferentiation or reprogramming into cancer-associated adipocytes that boost tumor energy supply and progression [61,62]. Other obesity–cancer association mechanisms include obesity-induced chronic inflammation [63,64]; increased aromatase expression, circulating levels of estrogen, insulin, leptin and ceruloplasmin; and decreased amounts of adiponectin and sex-hormone-binding globulin [61,65,66,67,68,69,70,71,72,73]. Estrogen, for instance, which can be produced by WAT fibroblasts, is a tumoral growth and malignancy activator through stimulating the expression of crucial receptors, such as the progesterone receptor (PR) and adenosine A1 receptor (ADORA1) [74], besides enhancing cellular multiplication and inhibiting apoptosis [65,75,76].

The implication of genetics in cancer might be prominently exemplified through the critical *BRCA1/2* tumor suppressor gene mutations, which, respectively, expose their holders to 60% and 30% higher risk of breast cancer [77,78,79] regardless of the woman’s menopausal status [67]. On the other hand, rare forms of obesity that are uniquely due to defects of genes encoding for, e.g., FTO, melanocortin 4 receptor or leptin, have also been identified [49,80,81,82]. Additionally, several studies have revealed the implication of obesity in strongly carcinogenic genomic instability [50,83]. This multi-branched and sophisticated obesity–cancer tie doubtlessly requires further interdisciplinary collaborative navigation across the genetic, epigenetic, and metabolic profiles of both diseases, and to be complemented also by deeper investigations into the implication of gut flora–host dishomeostasis [84,85,86,87].

## 4. Obesity and Cancer Common Features

### 4.1. Multifactorial Grounds and Deranged Metabolism

Both obesity and cancer are multifactorial, mainly caused by genetic predisposition, high-carbohydrate diet, and sedentary lifestyle, besides other etiologies that are being continually discovered and investigated, such as certain medications’ side effects or interactions [88,89], abnormal levels and impactful interactions of some sex hormones (such as estrogen and testosterone) [90,91,92,93], poor sleep habits [94], and stress-engendered pathways [95,96]. Although the genetic basis is best known as an origin of cancer, obesity also owes 40–70% of its interindividual variation to genetic grounds [97]. People should be sensitized to this so society can seriously act on the relatively more manageable factors, such as diet and physical exercise. However, this may not function for every person, as recent studies prove that certain individuals present an inherent state of baseline vulnerability towards obesity, which makes them non-respondent to weight-loss strategies such as low-energy diets [98] and further confirms the complexity of this disease.

Moreover, the predominance of the sedentary lifestyle during the current century, [99], promoted by cell phone (mobile) use, for example, has steered our attention away from our “cellular” health and how to stay “mobile”. This has intensified the positive energy balance and obesity levels within the population. Such a plurality of obesity origins should obviously trigger attentive caution. In fact, the term unifactorial disease is less and less utilized by scientists, as most morbi are rather classified into the multifactorial category. Even so, what is most featured in cancer and obesity, uncommonly to other morbi, is that they are also “multi-organ” diseases, where cancer cells as well as lipid depots might acquire a widespread existence throughout the organism. This multiplies the multifaceted challenging aspects of these diseases with regard to both their prevention and therapy.

It is also striking how deranged metabolism can hallmark both obesity and cancer. For example, high fructose consumption has been interrelated with tumoral growth and malignancy, as this sugar accelerates glycolysis, which fosters the Warburg effect through hastening aerobic fermentation and the production of lactate and uric acid, which are all in favor of cancer development and metastasis [100,101]. Interestingly, metabolic syndrome and obesity have also been suggested to be promoted by excessive fructose consumption [100,102,103]. As metabolism is a “cellular housekeeping process” that maintains whole-body functioning homeostasis, its disturbance in obesity and cancer is particularly intriguing because these morbi further disarrange cellular growth and proliferation.

### 4.2. Disordered Cell Growth and Proliferation

Obesity and cancer are both characterized by abnormal cellular growth and multiplication. The Dana–Farber comprehensive Cancer Treatment and Research Institution in Massachusetts justly defines cancer as “a disease in which cells, almost anywhere in the body, begin to divide uncontrollably. A tumor is when this uncontrolled growth occurs in solid tissue such as an organ, muscle, or bone. Tumors may spread to surrounding tissues through the blood and lymph systems. Cancer treatment aims to eradicate these abnormal cells, or to slow or stop them from spreading” [104]. Anomalous cell growth and proliferation are widely known fundamental aspects of cancer. Nonetheless, they are less perceived with respect to obesity pathways. One of the main physiological roles of WAT is fatty acid energy storage in the form of triglycerides in mature fat cells to prevent the circulation of these molecules throughout the bloodstream and their potential deposition in different ectopic locations. Additionally, at healthy adiposity levels, WAT plays its homeostatic physiological secretory roles to communicate with other organs—for example, by releasing the hormone leptin, which signals satiety to the brain, particularly the hypothalamus, as well as many other adipokines [105]. The physiological functions of WAT are preserved due to the healthy multiplication of preadipocytes; the progenitor cell reservoir that is ready to differentiate via adipogenesis into mature adipocytes that store lipids without a need for excessive cell growth or proliferation [106]. Nevertheless, obesity displays several alterations of these functions as mature fat cells undergo excessive hypertrophic growth, eventually resulting in cell death and triggering inflammation while preadipocytes proliferate excessively [107,108].

**Figure 1:**
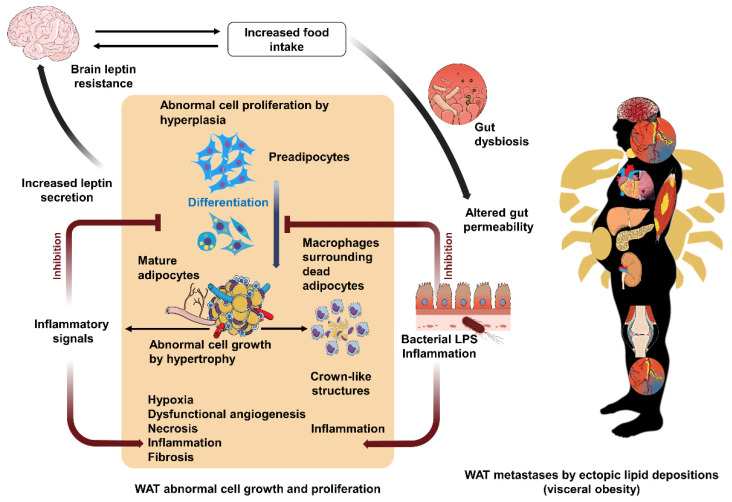
Vicious circles of white adipose tissue (WAT) abnormal cell growth and proliferation, and fat mass metastases. In obesity, the brain becomes less sensitive to the satiety hormone leptin, and this favors feelings of hunger and increases food intake. This further boosts the positive energy balance, induces intestinal flora dysbiosis and augments gut permeability to the bacterial endotoxin lipopolysaccharide (LPS), which is highly inflammatory. LPS inhibits the differentiation of preadipocytes to mature fat cells. Thus, more preadipocytes are formed to generate new adipocytes to store the body’s excess of energy, which enhances obesity by hyperplasia. The inhibition of adipogenesis induces mature adipocytes to undergo extreme abnormal cell growth to pack lipids that surpass their maximum storage capacity through excessive hypertrophy. This further causes hypoxia, fibrosis, and eventually cell necrosis. Massive WAT hypertrophy might induce severe hypoxia that can trigger angiogenesis, which, however, generates fragile and dysfunctional capillaries that further boost tissue inflammation. The dead adipocytes attract immune cells—macrophages—to form inflammatory crown-like structures. The affected WAT secretes high leptin levels to the brain to reduce food intake; however, the brain becomes insensitive to leptin due to the ongoing processes of obesity. WAT in the obese organism further secrets inflammatory cytokines to promote the inflammatory reaction in a chain of vicious circles that devastates WAT structure and function. This leads to lipid storage in other organs, like skeletal muscles and vital organs such as the heart, the liver, the kidneys, and the pancreas. Such expansion of WAT localization through ectopic fat depositions that embody “metastases” alters these organs functionality and therefore whole-body homeostasis. In addition, accumulated free fatty acids in blood circulation may injure endothelial cells through provoking oxidative and inflammatory reactions, which might hasten atherosclerotic plaque rupture. This can cause venous or arterial thromboembolism, which can present fatal consequences when affecting vital organs, such as in pulmonary embolism, heart attack, and stroke. These biological alterations are accompanied by those starting from a mechanical origin, such as body weight pressure on the bones and joints or airway blockage, to induce osteoarthritis or obstructive sleep apnea, respectively. These crab (cancer)-leg-like projections of cell growth, cell proliferation, and fat ectopic depositions, and their impact on the whole body in obesity, justly present high similarity with cancer disease metastases. Created with BioRender.com and with MindtheGraph.com. Accessed on 24 March 2022.

The WHO definition of obesity refers indirectly to these notions by defining it as the abnormal or excessive accumulation of WAT. This description indicates that there exists a balanced WAT amount in the absence of obesity [106]. Chronic, morbid positive energy balance alters WAT functionality, renders it insulin-resistant, and induces its immoderate secretion of leptin, resulting in a loss of sensitivity of the hypothalamus to this hormone in obesity [106]. This vicious circle further enhances food intake and lipid storage and aggravates the morbidity of adiposity. Indeed, in obesity, WAT is forced to highly multiply its number of preadipocytes in an endeavor to elevate the body’s ability to store lipids, especially when hypertrophic adipocytes reach their maximal capacity of growth, and die [106]. However, such proliferation further increases WAT mass via hyperplasia, which adds to that resulting from adipocyte hypertrophy [106]. This hyperproliferation can also be enhanced by obesity-induced gut dysbiosis that augments intestinal permeability and bacterial lipopolysaccharide (LPS) levels in the blood towards the WAT, where it can further increase this multiplication by inhibiting adipogenesis [109]. LPS, moreover, enhances WAT inflammation, which is already triggered by the crown-like structures formed by recruited immune cells, mainly macrophages, that surround dead hypertrophic adipocytes [109]. Such a chain of vicious circles promotes WAT morbid cellular growth and proliferation (Figure 1).

### 4.3. Metastasis

Consulting the Oxford Concise Medical Dictionary, we find that metastasis is “the spread of a malignant tumour from its site of origin”. Equivalently, the medically inexpert Oxford Learner’s Dictionary defines it as “the development of tumours in different parts of the body resulting from cancer that has started in another part of the body”. By browsing through the PubMed search engine, again we realize that the word metastasis is always linked to tumors or cancer. Thus, metastasis is mostly considered to be solely associated with cancer both for the public and for medical experts. This feature of cancer is its most feared because it multiplies the number of organs that are affected by the disease and magnifies its deadly effect. The definition of cancer by the WHO inherently includes this notion as it introduces cancer as the following: “Cancer is a large group of diseases that can start in almost any organ or tissue of the body when abnormal cells grow uncontrollably, go beyond their usual boundaries to invade adjoining parts of the body and/or spread to other organs. The latter process is called metastasizing and is a major cause of death from cancer. A neoplasm and malignant tumour are other common names for cancer”.

These definitions show tight similarity with ectopic lipid deposition in obesity, where the WAT goes beyond its usual limits, which are mostly subcutaneous, to infiltrate other organs through the uncontrolled expansion of visceral WAT [110]— a depot that, like in cancer metastases, has been suggested to be functionally and metabolically different from healthy deposits [111,112]. When visceral WAT also becomes insufficient to store excess triglycerides, these end up being deposited not only in skeletal muscles, but also in the liver and other vital organs—such as the kidneys, pancreas, and heart—whose biological machinery is not suited to store lipids, and, therefore, eventually become dysfunctional to the point of complete organ failure [110,113]. In addition, the accumulated free fatty acids in the plasma may injure endothelial cells via triggering oxidative stress and inflammatory reactions, which may prompt atherosclerotic plaque rupture [114]. The migrating thrombus might cause venous or arterial vessel thromboembolism that can be lethal, such as in pulmonary embolism, myocardial infarction, and stroke [115]. Therefore, such fat metastases can lead to whole-body dishomeostasis (Figure 1). In summary, obesity, like cancer, is distinctive due to the danger of “metastases” when compared to other diseases that target mainly one organ. This concept can be extended even further if one considers that, although not necessarily via diffusion of fat in the body: (1) obesity is very often followed by type 2 diabetes (which is as a dysfunction of the pancreas), which in turn can lead to the failure of several peripheral organs and tissues [116], and (2) obesity is accompanied by neuroinflammation and alterations in synaptic plasticity, thus also affecting brain function [117,118].

### 4.4. Aggravation by Angiogenesis

Healthy forward and backward blood vascularization is key for the proper functioning of body tissues. Such a crucial input and output system is so necessary that capillary blood vessels can burgeon from the primary ones in response to tissue growth requirements through a process known as angiogenesis [119]. However, diseased tissues can also utilize such a sprouting strategy to grow, as best known in cancer [120]. Indeed, most research publications and therapeutical applications of angiostatic mechanisms are dedicated to anticancer treatment, for instance, through combining them with other interventions [121,122]. However, angiogenesis is implicated in many other common diseases, such as arthritis, polycystic ovary syndrome, asthma, and obesity [120].

In fact, WAT possesses rich blood vascularization that supports its multiple functions, and angiogenesis is one of its physiological growth mechanisms, besides hyperplasia and hypertrophy, which both can signal to enhance it [123,124]. In healthily growing WAT, angiogenesis is stimulated to compensate for the minor hypoxia resulting from mild WAT mass increase [125]. In contrast, the considerably expanded WAT size in obesity, through massive hypertrophy, results in serious tissue hypoxia, which the tissue vascularization cannot cope with [106]. In addition, this excessive hypoxia might lead to WAT fibrosis and inflammation and can eventually induce necrosis processes [106]. Furthermore, obesity-associated WAT inflammation drives and is enhanced by the growth of unhealthy capillaries, whose features are fragile structural remodeling and leakiness, that infiltrate the WAT with immune cells and boost its inflammation [126]. These pathological processes are exacerbated by the synergistic activation of inflamed WAT intrinsic immune cells with those that infiltrate the WAT, such as macrophages, which both release inflammatory secretions to further stimulate unhealthy capillary growth and add a further vicious circle to these tissue-diseasing processes [122,123,127,128].

Oncology research has shown that multiple angiogenic proteins and their receptors show increased expression triggered by cancer-related hypoxia and inflammatory cytokines, such as tumor necrosis factor alpha (TNF-α) and interleukin-6 (IL-6). One important such protein is the vascular endothelial growth factor (VEGF) [129,130]. Cancer-cell-secreted VEGF binds its receptor on the surface of endothelial cells to promote capillary growth that, in turn, nourishes cancer growth [129]. Likewise, WAT is richly vascularized with its inherent endothelial cells that communicate paracrinally with their “tissue-mate” fat cells, which can secrete similar proteins to those expressed in cancer—such as TNF-α, IL-6, and VEGF—and also antiangiogenic factors, such as endostatin, which—while balanced in healthy WAT—might indirectly or directly exacerbate this tissue hypoxia in obesity through inducing ineffective or repressed angiogenesis [124,131,132]. This might result in further exacerbation of the aforementioned tissue-damaging vicious circles that bring about hypoxia and inflammation. Additionally, aberrant angiogenesis impairs WAT communication and signal transmission to other organs and systems, which is a concrete endocrine role that WAT must play to maintain whole-body homeostasis [133].

However, targeting neovascularization to treat obesity is still in its very early stages compared to treating cancer [134]. For example, angiostatin is already undergoing clinical trials to treat human cancer [135], and endostatin is another antiangiogenic drug that is already an antineoplastic medication [136]. Obesity research might, therefore, follow and learn from the use of angiostatic statins against cancer.

The metastatic development of obesity and cancer presents a unique pathological networking strategy of disease-spreading throughout the body, as explained in the previous subsection. This networking escalation—by hijacking the functions of the body’s largest network, i.e., the vascular system, and burgeoning new, but unhealthy, blood vessels that nurture these diseases’ unhealthy growth, hypoxia, and inflammation—can directly further aggravate the progression of these disorders [120].

### 4.5. Progressive Development and Stages

One of the reasons why people may consider obesity as a less serious illness than cancer is that they compare advanced stages of cancer to “benign” obesity stages. In fact, both illnesses might advance progressively in the absence of proper diagnosis and intervention and may subtly develop until suddenly triggering stroke [137] or an advanced brain tumor [138], for instance. However, deaths that are caused by obesity are massively attributed to the consequent morbi and neglect the implication of obesity, such as in cardiovascular ischemic events or even cancer. On the contrary, cancer is always indicated as the death cause when it is involved. Obesity is globally classified into three categories based on BMI measurements, with respect to certain adaptations according to ethnicity and athlete or pregnant status, namely, obesity class I—BMI 30 kg/m^2^ to 34.9 kg/m^2^; obesity class II—BMI 35 kg/m^2^ to 39.9 kg/m^2^; and severe obesity (class III) for higher BMI values [139]. The more recent Edmonton Obesity Staging System (EOSS) ranges obesity stages from zero to four, in accordance with the occurrence of related comorbidities in addition to the overall health situation, where stage 4 is the severe, potentially end-stage [140] that might be properly labeled “malignant”, with reference to its seriously damaged health profile.

Likewise, tumor progression is classically divided into four classes considering its growth characteristics and propagation, namely, classes I (A, B, C), II, III, and finally class IV lesions marked with metastases [141]. The more updated tumor node metastasis (TNM) classification system based on recent advances in cancer research has been internationally recognized as the optimal criterion for cancer staging by involving tumor size and local growth (T), the magnitude of lymph node metastases (N), and the existence of distant metastases (M) [142]. Similar classifications might be suggested for obesity, to measure the extent of visceral deposits. In summary, both obesity and cancer present stages of progression and thus display categories of illness seriousness from “benign” to “malignant”. However, these descriptions are rarely used when referring to obesity. The phrase “malignant obesity” could properly warn of its peril.

### 4.6. Recurrence

The fear of cancer recurrence has been extremely heeded and scrutinized. This bestowed it with the 42-item Fear of Cancer Recurrence Inventory (FCRI) scale, which was developed to assess its severity [143]. Obesity can also present relapses, named weight cycling or yo-yo effect, which refers to unintentional and out of control weight regain and its maintenance [144]. However, when consulting the literature and obesity terminology, we fail to notice a scale that evaluates the fright of obesity. What we find is the 50-item Fat Phobia Scale, for example, which does not examine the fear of obesity recurrence, nor even of the obesity disease per se [145]. Instead, it estimates the feelings and judgments towards people suffering from obesity [145]. Even the updated versions of the Fat Phobia Scale did not add measurements of obese patients’ fear of their condition or its relapse, but rather were limited to shortening the scale form under the same old concept [145,146]. Obviously, a lot of work is needed to properly address obesity relapse. This can start by learning from cancer recurrence scales, such as by establishing one named Fear of Obesity Recurrence Inventory. This might concede and evaluate the fear of obesity relapse to better help approach the cases in which patients are frightened of becoming stuck in a recurrent obese status. Such endeavors could aid in the improvement of population awareness about such a potentially recurrent morbus that should be “feared” instead of the patients with obesity.

### 4.7. Multidimensional Complexity from the Gut Microbiome

An increasing body of evidence supports the existence of a sturdy link between the micro-organisms living in our gastrointestinal system and constituting, with their genes and their armamentarium of bioactive molecules, the gut microbiome, and their host health or disease status [147]. This association is specifically fateful in obesity and cancer [148,149] due to the synergistic combination of these diseases unique complex features, explained above, and the huge intricacy of the gut microbiota community that is still being deciphered [150]. Being both metastatic and chronic disorders, obesity and cancer can devastate body functions in terms of both the dimension of body area and that of time of progression. Their impact is mostly further complicated by their playing on a third, very sophisticated, dimension, i.e., that of the gut microflora. Several recent reports have highlighted that the gut microbiota do not merely play one major role in cancer and obesity pathology. In fact, they can play several roles, i.e.,: (i) by presenting potential transmissibility of the disease but also of a healthier status through therapeutic fecal microbiota transplantation [151,152]; (ii) by providing opportunities for nonconventional therapeutic strategies, such as probiotic, prebiotic, or symbiotic treatments [153,154]; and (iii) by acting as both an actor and biomarker of the host’s response to the treatment [155,156]. This tridimensional complexity that combines time, space, and gut microbiome is more perplexing if one considers the other dimensions that affect the gut microbiota ecosystem, such as genetics, epigenetics, and developmental and environmental/lifestyle factors [157,158].

## 5. Conclusions and Perspectives

Obesity is a disease by scientific definition and international organization recognition. It further shares a plethora of morbidity characteristics with the most feared illness worldwide, cancer. This is because (1) it has multiple causes, (2) it presents anomalous cell growth and proliferation, (3) it displays metastatic events, (4) it can unhealthily develop through angiogenesis, (5) it might advance progressively from a “benign” to a “malignant” stage, (6) it has a high risk of relapse, and (7) it can present multidimensional complexity from the gut microbiome. Moreover, obesity contributes to the onset and deleterious progress of several other serious morbi, including those of more than 13 types of cancer [12]. Thus, very strong similarities and connections exist with cancer. Finally, the development, progress, and response to therapy of both obesity and cancer are now considered to also be strongly influenced by the gut microbiome. Despite these facts, many people doubt the disease identity of obesity, which imposes the urgent need for more population sensitization about its dangers.

This can be achieved by correcting our obesity terminology to rectify our unconscious beliefs that can be deeply affected by our words [159,160,161]. We can encourage others to stop using the words “lose” and “gain” when describing body weight changes. Such words, besides employing the term deposit, when referring to obesity, may indirectly refer to our hidden desire to have extra weight and fat depots as we want extra money and its deposit. Has anyone had heard phrases like “lose” or “gain” a tumor? Similarly, the term “metastasis” could more suitably indicate the gravity of ectopic lipid deposition instead of the word “deposit”. Moreover, the word “yo-yo” should be replaced by the more serious designation “recurrence”, which seriously emphasizes that obesity is far from being a game. Likewise, the word “neoplasia”, which means new formation (from Greek), which is solely used to refer to tumoral growth, can be further utilized to appropriately describe ectopic fat establishment in visceral obesity, which indeed results from neo-fat-mass growth. These propositions can be applied in scientific publications and presentations and to refine official health organizations’ and associations’ definitions of obesity. To dispel any doubts, this serious pathology should be directly named “obesity morbus” to engrave this identity of it in our minds, attitudes, and actions. We hope that this multioriented vigilance weathervane will inspire optimized navigation through the perception of other diseases as well.

## Data Availability

Not applicable.
